# Current Research on Zinc Oxide Nanoparticles: Synthesis, Characterization, and Biomedical Applications

**DOI:** 10.3390/nano12173066

**Published:** 2022-09-03

**Authors:** Ashok Kumar Mandal, Saurav Katuwal, Felix Tettey, Aakash Gupta, Salyan Bhattarai, Shankar Jaisi, Devi Prasad Bhandari, Ajay Kumar Shah, Narayan Bhattarai, Niranjan Parajuli

**Affiliations:** 1 Natural Product Research Laboratory, Thapathali, Kathmandu 44600, Nepal; 2 Central Department of Chemistry, Tribhuvan University, Kirtipur 44618, Nepal; 3 Department of Chemical, Biological, and Bioengineering, North Carolina A&T State University, Greensboro, NC 27411, USA; 4 Department of Chemistry and Biochemistry, University of Massachusetts Dartmouth, North Dartmouth, MA 02747, USA; 5 Paraza Pharma, Inc., 2525 Avenue Marie-Curie, Montreal, QC H4S 2E1, Canada; 6 Faculty of Health Sciences, School of Health and Allied Sciences, Pokhara University, Lekhnath 33700, Nepal

**Keywords:** zinc oxide nanoparticles, green synthesis, biological activities

## Abstract

Zinc oxide nanoparticles (ZnO-NPs) have piqued the curiosity of researchers all over the world due to their extensive biological activity. They are less toxic and biodegradable with the capacity to greatly boost pharmacophore bioactivity. ZnO-NPs are the most extensively used metal oxide nanoparticles in electronic and optoelectronics because of their distinctive optical and chemical properties which can be readily modified by altering the morphology and the wide bandgap. The biosynthesis of nanoparticles using extracts of therapeutic plants, fungi, bacteria, algae, etc., improves their stability and biocompatibility in many biological settings, and its biofabrication alters its physiochemical behavior, contributing to biological potency. As such, ZnO-NPs can be used as an effective nanocarrier for conventional drugs due to their cost-effectiveness and benefits of being biodegradable and biocompatible. This article covers a comprehensive review of different synthesis approaches of ZnO-NPs including physical, chemical, biochemical, and green synthesis techniques, and also emphasizes their biopotency through antibacterial, antifungal, anticancer, anti-inflammatory, antidiabetic, antioxidant, antiviral, wound healing, and cardioprotective activity. Green synthesis from plants, bacteria, and fungus is given special attention, with a particular emphasis on extraction techniques, precursors used for the synthesis and reaction conditions, characterization techniques, and surface morphology of the particles.

## 1. Introduction

A diverse application of nanomaterial-based technology has opened a new horizon in material science over the past decades because nanomaterials offer a high surface area and other very distinctive physical, chemical, and biological properties compared to their bulk counterparts [1]. Nanoparticle (NP) research has gained distinct interest due to the enhanced electrochemical reactivity, thermal conductivity, and nonlinear optical properties of nanoparticles which offer unique applications [2]. Zinc oxide nanoparticles (ZnO-NPs) are the most commonly used metal oxide nanoparticles because their distinctive optical and chemical properties can be easily modified by altering the morphology and the wide bandgap (3.37 eV) and high excitation binding energy (60 meV) to simulate the ZnO-NPs to be a potent photocatalytic and photo-oxidizing moiety against chemical and biological species [3,4]. They are less toxic to the human body and offer biocompatibility as the Zn ion (Zn^2+^), a soluble form of ZnO, is a trace element found in the human physiological system. ZnO-based structures have been proven to exhibit biodegradability both in the bulk phase and in the form of nanoparticles [5]. Zn ions also act as the principal mediators of intracellular bacterial toxicity, disrupting their cell membranes [6].

Some potential applications where ZnO-NPs have been researched are: therapeutic carriers, biological sensing, gene transfer, nanomedicine discovery, biological labeling, medical implant coatings, electronic sensors, wastewater treatment, and communication [4,7,8]. The medical implant coating with zinc oxide and hydroxyapatite exhibited antibacterial and osteoconductive properties, emphasizing the potential of ZnO-NPs in therapeutic diagnostics. ZnO-NPs exhibited cytotoxicity in human cancer cells, resulting in cell death via the apoptotic pathway [9]. They also promoted antiproliferative activity in triple-negative breast cancer cells [10], nonautophagic cell death in human lung adenocarcinoma cells with an epidermal growth factor receptor (EGFR) mutation [11], and anticancer activity via apoptosis in chronic myeloid leukemia cells using a transcriptomic approach [12]. It has also been shown to induce cytotoxicity in the A549 epithelium and cancer cells [13]. Recent investigations on the ZnO-Au nanocomposite have developed an electrochemical DNA biosensor [14], ZnO-NPs for tracing studies in plants [15], and material in the development of electrochemical sensors in the detection of food additive aspartame [16]. ZnO-NPs have been shown to influence horizontal gene transfer where it impacts the transformation efficiency of *Bacillus subtilis* [17], and the ZnO-Ag NPs have decreased the rate of biofilm formation and gene expression in *Staphylococcus aureus* at a subminimum inhibitory concentration [18]. ZnO-NPs have been shown to reduce the parameters responsible for hepatic fibrosis (hydroxyproline) and nephrotoxicity (creatinine, urea, and uric acid) [19], also attenuating the gonadal toxicity which is induced by cyclophosphamide (an anticancer and immunosuppressant drug) through their antioxidant and antiapoptotic function [20], and cancer cell death through autophagy induction which supports the release of zinc ions and the generation of reactive oxygen species (ROS) [21].

In a critical study, zinc ions and ZnO-NPs both showed cytotoxic effects in the earthworm GI tract where it affected the gut epithelium and chlorogenic tissues [22]. However, ZnO-NPs dissolve slowly in human physiological conditions (pH 6–8), and the United States Food and Drug Administration (USFDA) safety datasheet indicates ZnO as a “Generally Recognized as Safe” (GRAS) substance and nonhemolytic against human red blood cells [23]. ZnO could be discovered to be a useful nanocarrier to facilitate the drug-delivering and release processes [24,25]. Much research endorses ZnO-NPs as the most beneficial metal nanoparticles, with minimal toxicity and excellent biocompatibility. The structural atom allocation mimics the most bioactive agent, emphasizing its pharmacological effectiveness against various ailments. With all this potential, the objective of this review article is to explore the various synthesis approaches and characterization techniques of ZnO-NPs with a comprehensive mechanistic approach to its biological activity. Although there is an increased number of studies revealing the mutually exclusive and exhaustive area of ZnO-NPs, this review is a comprehensive compilation of recent advances with clear illustrations for a better understanding of the importance of ZnO-NPs in biomedical research.

## 2. Biological Activities of ZnO-NPs

### 2.1. Antibacterial Action of ZnO-NPs

**Figure 1:**
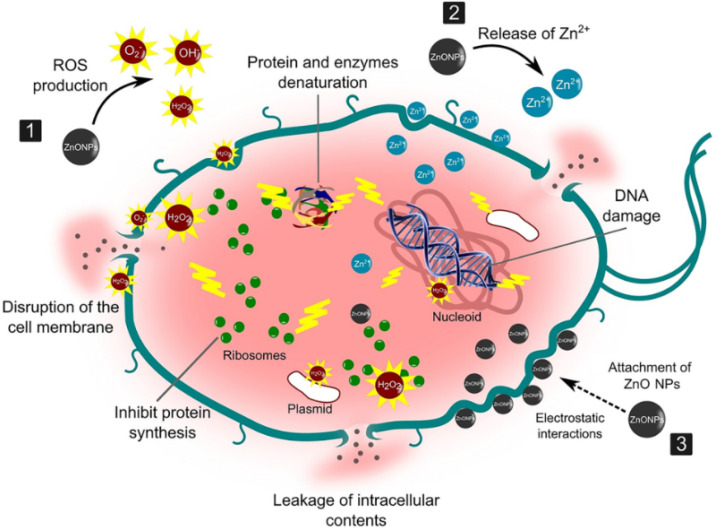
Illustration of the antimicrobial property of ZnO-NPs against the bacterial cell wall. They act as potent antibacterial agents through these possible steps: (1) production of reactive oxygen species (ROS) causing oxidative stress, and membrane and DNA damage leading to bacterial death; (2) dissolution of ZnO-NPs into Zn^2+^ and interference with bacterial enzymes, proteins, and amino acids; and (3) electrostatic interaction between ZnO-NPs and cell membrane, resulting in membrane plasma damage and intracellular content leakage. (Reprinted from [29]; open access under CC BY).

**Figure 2:**
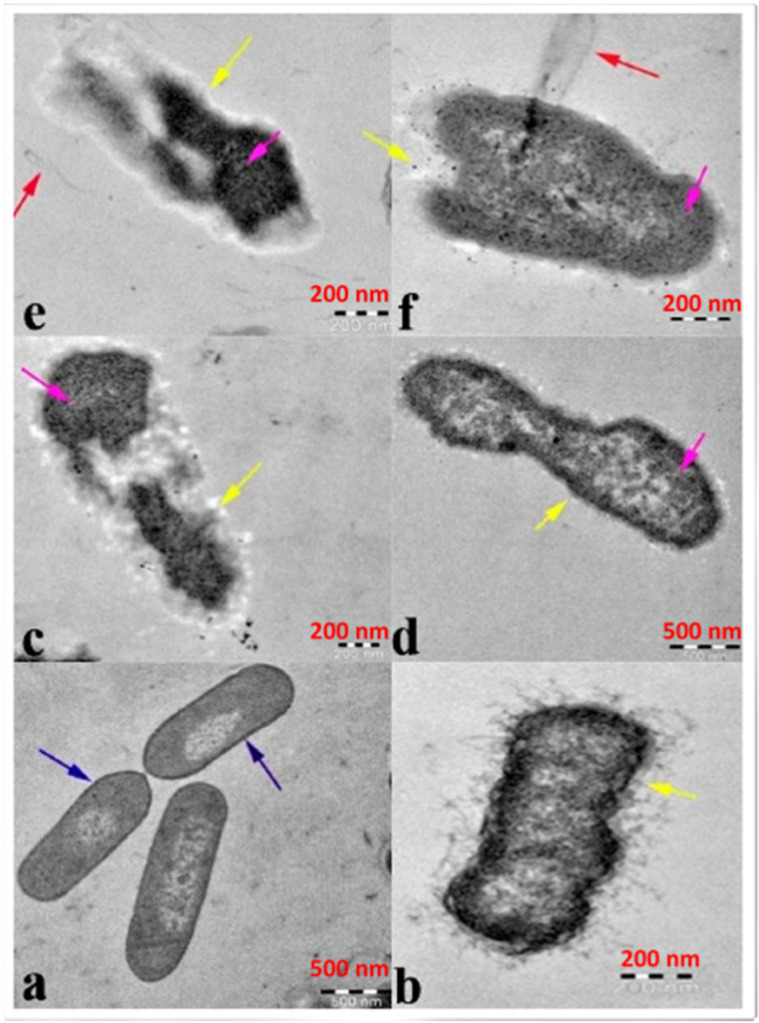
Image illustrating antibacterial efficacy against β-lactam-resistant *K. pneumoniae* obtained using transmission electron microscopy: (**a**) ZnO-NPs in the untreated state and ZnO-NPs in the treated state (**b**–**e**). Cytoplasmic shrinkage (**b**) disrupted cell wall and membrane (**c**), denatured protein shows as a dark electron-dense patch (**d**), and cytoplasmic spillage (**e**,**f**). The blue arrow represents an intact cell wall, the yellow arrow represents a disintegrating cell wall and cell membrane, and the violet arrow represents a denatured protein. (Reprinted from [30]; open access under CC BY).

Bacteria portray a severe threat to human life as the world grapples with escalating antibiotic resistance and bacterial infection. ZnO-NPs have remarkable photo-oxidation and photocatalytic characteristics, and their exceptional antimicrobial properties have led to their recognition as potent agents against MDR [26]. Although the mechanism of antimicrobial action of ZnO-NPs is not well established, its properties, such as zinc ions and ROS generation, are widely assumed to result in oxidative stress and DNA damage, as well as photocatalytic activity, contributing to antibacterial efficacy (Figure 1). According to Sirelkhatim et al., the oxygen annealing of ZnO increases the number of oxygen atoms on the surface, resulting in increased oxygen atom adsorption and the generation of more ROS, resulting in enhanced oxidation, and hence, a facilitated antimicrobial property [27]. Moreover, ZnO-NPs cause cytoplasmic shrinkage and the disruption of cell walls leading to cytoplasmic spillage (Figure 2). ZnO-NPs act as an effective bactericidal agent against both Gram-positive as well as Gram-negative bacteria and are found to have direct interaction with the cell wall of bacteria leading to the disruption of its integrity [28].

### 2.2. Antifungal Action of ZnO-NPs

The antifungal properties of ZnO-NPs have been discovered in various studies in the literature. Their fungicidal activity varies depending on their structure, size, and concentration. The antifungal potency of biofabricated ZnO-NPs against *Candida albicans* isolates was investigated, and it was revealed that they were more effective against drug-resistant *C. albicans* isolates, demonstrating ZnO-NPs’ antifungal potency. Furthermore, it was shown that prophylactic treatment with lower concentrations of ZnO-NPs protects *G. mellonella* from the infection of *C. albicans* [31,32]. Similarly, the antifungal resistance of a 2% ZnO-NP-based cold cream exceeded the activity compared to a commercial antifungal cream at 2% on clinical isolates of *Candida* sp. [33]. ZnO-NPs have antifungal activity against both *Aspergillus* and *Penicillium* and have been investigated for their antidermatophytic activity on *Trichophyton mentagrophytes* and *Trichophyton verrucosum* [34,35]. Likewise, the bionanocomposite film of the soy protein isolate (SPI), cinnamaldehyde (CIN), and ZnO-NPs exhibited the highest antifungal activity among SPI, SPI-CIN, and SPI-ZnO-NPs films, where it was 1.56-fold stronger compared to the SPI-ZnO film and 1.24-fold stronger compared to the SPI-CIN film [36]. The antifungal activity studied against two pathogenic fungi—*Botrytis cinerea* and *Penicillium expansum*—revealed that activity is also dependent on nanoparticle concentrations, with the efficacy of the ZnO-NP treatment increasing as the concentration of ZnO-NPs rose from 3 to 12 mM. By affecting cellular functions, ZnO-NPs cause deformation in fungal hyphae, inhibiting the growth of *B. cinerea*. Similarly, *P. expansum* prevents the formation of conidiophores and conidia, resulting in the death of fungal hyphae, explaining the fact that *P. expansum* is found to be more sensitive than *B. cinerea*, i.e., microbe dependent. The activity detected in *B. cinerea* revealed the stronger the photo-activation, the greater the activity [37,38,39].

### 2.3. Cytotoxic Effect of ZnO-NPs

**Figure 3:**
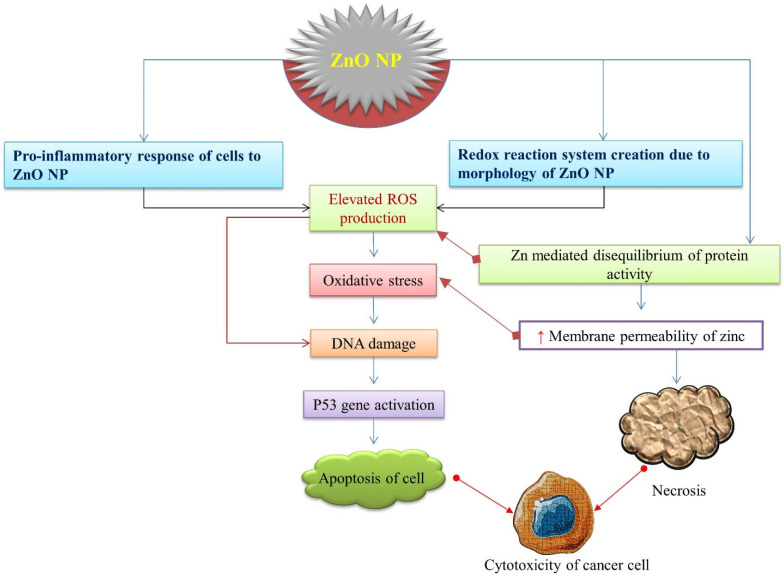
A schematic representation of cytotoxicity potency of ZnO-NPs leading to the death of cancer cells. ZnO-NPs induce ROS production sequentially, leading to oxidative stress, DNA damage, p53 activation, and apoptosis of cancerous cells.

ZnO-NPs, compared to other metal oxide NPs, have a significant effect on cancer cells. The anticancer potential of ZnO-NPs is strongly influenced by their shape, size, and concentration. It has been discovered that the smaller the size and higher the concentration of NPs, the greater the anticancer activity [40,41]. They showed concentration-dependent anticancer activity against MCF7 human breast cancer cells, where 93% inhibition of proliferation of cells was noted at 100 µg/mL [40]. Similarly, fabricated ZnO-NPs exhibited concentration-dependent growth inhibition in human pancreatic cancer cell lines, PNAC-1, and AsPC-1, although they were shown to have a relatively smaller effect on the human normal fibroblast cell line (Hu02), which was found by an MTT assay [42]. The mechanistic approach (Figure 3) underlying its anticancerous activity includes the production of sufficient ROS to cause substantial oxidative stress and DNA damage, disturbances on lipids and proteins in cells, and other cellular components due to their large semiconductor band gap [43]. Moreover, the establishment of a redox reaction system and the pro-inflammatory response of cells against ZnO-NPs induce cellular apoptosis. Discrimination between cancerous and normal cells has been a major challenge for a drug to be categorized as anticancerous. Failure to achieve selectivity results in systemic toxic effects. Several studies have revealed the selectivity of ZnO-NPs toward cancerous cells. ZnO-NPs have been demonstrated to be selective to Jurkat cancer cells with minimal toxicity toward normal CD4^+^ T cells [44]. Similarly, Hanley and the group proposed that ZnO-NPs had 28–35 times the specific cytotoxicity against cancer carcinoma cells compared to normal cells [45]. Selective localization by enhanced permeability and retention (EPR) time via extravasation toward tumor cells assists in selective activities affecting tumor cells rather than the normal cells. The electrostatic property of ZnO-NPs facilitates the targeting of tumor sites [46]. Thus, there is ample evidence that ZnO-NPs can exhibit anticancer effects in specific types of tumor cells in the body, which is depicted in Figure 3.

Despite various biomedical applications such as anticancer therapy, drug delivery, gene therapy, and tumor imaging, ZnO-NPs might have deleterious effects on several key organs including the lungs, kidneys, liver, CNS, reproductive system, and fetal development in animal models. However, the ZnO-NP-induced toxicity is multifactorial, and it is yet unknown just how toxic ZnO-NPs are for these organs [47].

### 2.4. Wound Healing Activity of ZnO-NPs

Wound healing is the phenomenon of cell injury responses, involving the activation of fibroblasts, endothelial cells, and macrophages where fibroblasts proliferate; an important step in wound healing for tissue regeneration [48]. It has been predicted that the delivery of ZnO via poly (lactide-co-glycolic acid) (PLGA)/silk fibroin (SF) nanofibers retains the bioavailability of NPs on the wound area and integrates with the unique structural features of electrospun nanofibers, which stimulate wound closure, re-epithelialization, collagen deposition, cellular migration, and angiogenesis [49]. Besides this, the ZnO-NPs loaded on bromelain-immobilized silk fibroin (SF-Br) reduced inflammation and promoted wound healing on a second-degree burn dressing [50]. During the healing process, the low doses of ZnO-NPs favored attachment and proliferation of fibroblasts, but the trend reversed at high doses. Metallic particles in nanocrystalline forms reduce wound infection along with promoting wound healing, as observed in adult male albino Wistar rats [51] and albino rats [52]. It was found that the functionalization of ZnO-NPs into triethoxysilane poly(amidoamine) dendrimer to generate a cross-linked collagen scaffold enhances re-epithelization and speedier collagen deposition than other scaffolds, which resulted in instantaneous wound healing [53]. In addition, the biodegradable thiolated bandage with implanted ZnO-NPs demonstrated an enhanced therapeutic agent for treating surgical site infections, satisfying the criteria for the optimal surgical dressing [54].

Similarly, the functionalization of bacterial nanocellulose (BNC) grafted with aminoalkyl silane and doped with Pullan-ZnO-NPs electrospun nanofibers (A-g-BNC/Pul-ZnO) exhibited superior performance in blood clotting and antibacterial activity that had a 5 log value higher than BNC, and was found to be safe in terms of cytotoxicity as tested in L929 fibroblast cells. It offers growth and proliferation, which was corroborated by the rat model where the scaffolds revealed rapid wound healing due to re-epithelization, and blood vessel and collagen formation [55]. An in vitro study reported that the bionanocomposite-based 3D chitosan/pectin/ZnO-NP porous films demonstrated no cytotoxicity (biocompatibility) and cell growth and migration (proliferation) for primary human dermal fibroblast cells (HFCs), suggesting a benign biomaterial for promoting wound healing [56].

Moreover, 3D-printed alginate-ZnO-NP hydrogels exhibited enhanced pore sizes, stiffness, and no detrimental effect on STO-fibroblasts or cell viability, making them a suitable scaffold for wound healing [57]. Generally, hydrogels are preferred with ZnO-NPs because they have a slow release of nanoparticles from the preparation, which reduces the cytotoxicity from ROS formation and improves wound healing. The above analyses support the findings of Saddik et al., where it was demonstrated that azithromycin-ZnO-NPs impregnated into an HPMC gel enhanced bacterial clearance and epidermal regeneration, which eventually stimulated tissue formation, leading to the rapid healing of the infected wound [58,59]. Another bioscaffold made from sodium alginate gum acacia ZnO-NP hydrogels showed a similar potential in expediting healing in terms of reducing inflammation and produced no scar at the excision wound on rabbit skin [60]. Thus, topical zinc application has been shown to improve the process of re-epithelialization, reduce inflammation, and inhibit the growth of bacteria in the case of foot ulcers and other topical wounds [61].

### 2.5. Anti-Inflammatory Activity of ZnO-NPs

**Figure 4:**
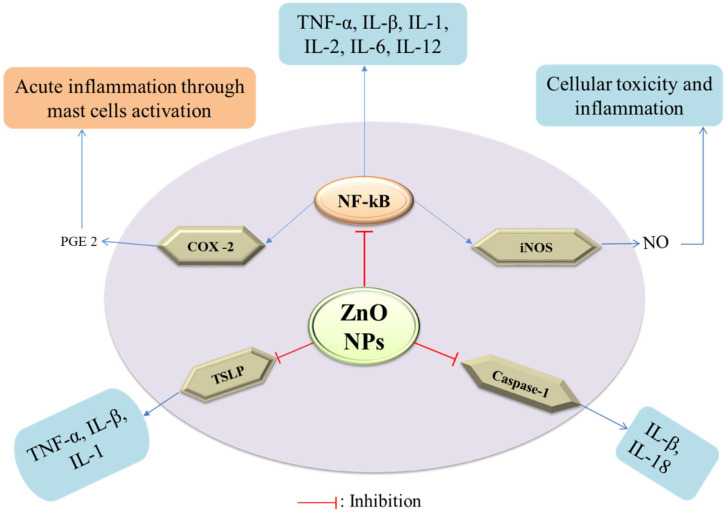
Mechanism of anti-inflammatory potency of ZnO-NPs.

The inflammatory response in the human body is a complicated process that involves immune system activation and the release of pro-inflammatory cytokines such as interleukin (IL)-1, -6, -12, -18, TNF-α, INFγ, and granulocyte-macrophage colony-stimulating factor (GMS-CF) [62] (Figure 4). Nuclear factor-kappa b (NF-κβ) is a key transcription factor that regulates the expression of many genes that encode pro-inflammatory mediators, such as COX-2 and iNOS, which increase the synthesis of pro-inflammatory mediators such as PGE2 and nitric oxide [63]. The ZnO-NPs act as anti-inflammatory agents as they have been shown to inhibit the release of pro-inflammatory cytokines, inducible nitric oxide synthase (iNOS) expression, myeloperoxidase, the NF-κβ pathway, and mast cell degranulation [64]. The mRNA expression of pro-inflammatory cytokines was suppressed by the ZnO-NPs synthesized using Polygala tenuifolia in a dose-dependent manner [65]. In addition, ZnO-NPs, when doped with aluminum, have been shown to reduce the production of thymic stromal lymphopoietin (TSLP) and caspase-1 activation in mast cells, leading to lowering the expression of pro-inflammatory cytokines, IL-1, IL-6, and TNF-α [66]. In a comparative study of ZnO-NPs and the ZnO standard form, it was revealed that ZnO-NPs relatively lowered the carrageenan-induced paw edema and amplified the anti-inflammatory activity of the nonsteroidal anti-inflammatory drug, ketoprofen, when administered intraperitoneally [67]. However, both forms were ineffective when administered per os (po) and guarded the gastric mucosa against the gastric ulcer induced by the administration of ketoprofen. ZnO-NPs have been discovered to have an excellent capping of flavones such as isoorientin, orientin, isovitexin, and vitexin, which have a potent anti-inflammatory response in a variety of ways, including the inhibition of cyclooxygenase, phospholipase A2, and lipoxygenases (enzymes that produce eicosanoids), resulting in a decline in leukotrienes and prostanoids [68].

### 2.6. Orthopedic Implants and Bone Healing Activity of ZnO-NPs

**Figure 5:**
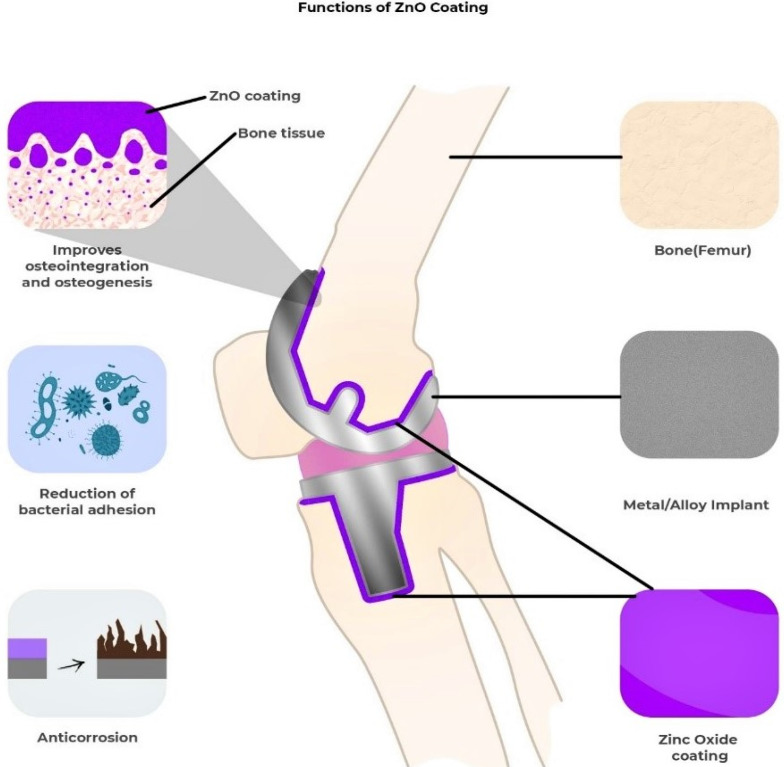
A diagram showing the effects of metal oxide (e.g., ZnO) coating on the orthopedic implant and bone.

Diseases such as osteoporosis, arthritis, and fibrous dysplasia can cause bone abnormalities and lasting disability. The implantation of orthopedic implants and scaffolds has significantly aided in the treatment of these bone diseases and abnormalities since they consist of materials with positive effects on the bone regeneration process [69]. Orthopedic implants are usually made of metals and alloys such as titanium, nitinol, stainless steel, and Co-Cr alloys [70]. Over the last several decades, these metals have been excessively utilized for deformity correction, joint replacements, fracture fixation, soft tissue anchorage, and most importantly, for accelerating bone growth [71]. Unfortunately, orthopedic implants are not free from side effects once placed in the body, leading to infections, limited corrosion resistance, low cell proliferation, excessive inflammation, and poor osseointegration [72,73]. If infection occurs, the implant loosens, bones take longer to heal, and sometimes prolonged suffering leads to death [74]. If corrosion occurs, toxicity incites, weakening the implant [70]. Metal oxide nanoparticles such as ZnO, magnesium oxide (MgO), iron oxide, zirconium oxide, titanium oxide, and silver oxide, when used with orthopedic implants, provide a wide range of solutions for the issues mentioned earlier. Figure 5 highlights how the ZnO coating on the implant helps in osteointegration, the prevention of biofilm formation, and the prevention of premature corrosion of the implant.

Biodegradable metals (BMs) such as Zn, Mg, Ca, and Fe have additional desirable properties for their applications in orthopedics [75,76]. During biodegradation, these metals release metal ions, metal oxides, and hydroxides. The close interaction between the degraded by-product and the stem-progenitor cells at the interface is what gives bone tissue implants their bioactivity [77]. Therefore, altering the implant’s chemical composition can have a significant impact on the treatment’s effectiveness [77]. The integration of growth factors into bone tissue scaffolds and implants is a prominent area of interest in the research. Protein growth factors such as insulin-like growth factors and bone morphogenetic proteins can activate cellular signaling cascades to stimulate active healing [78], including angiogenesis, a crucial step in bone tissue regeneration [79].

**Figure 6:**
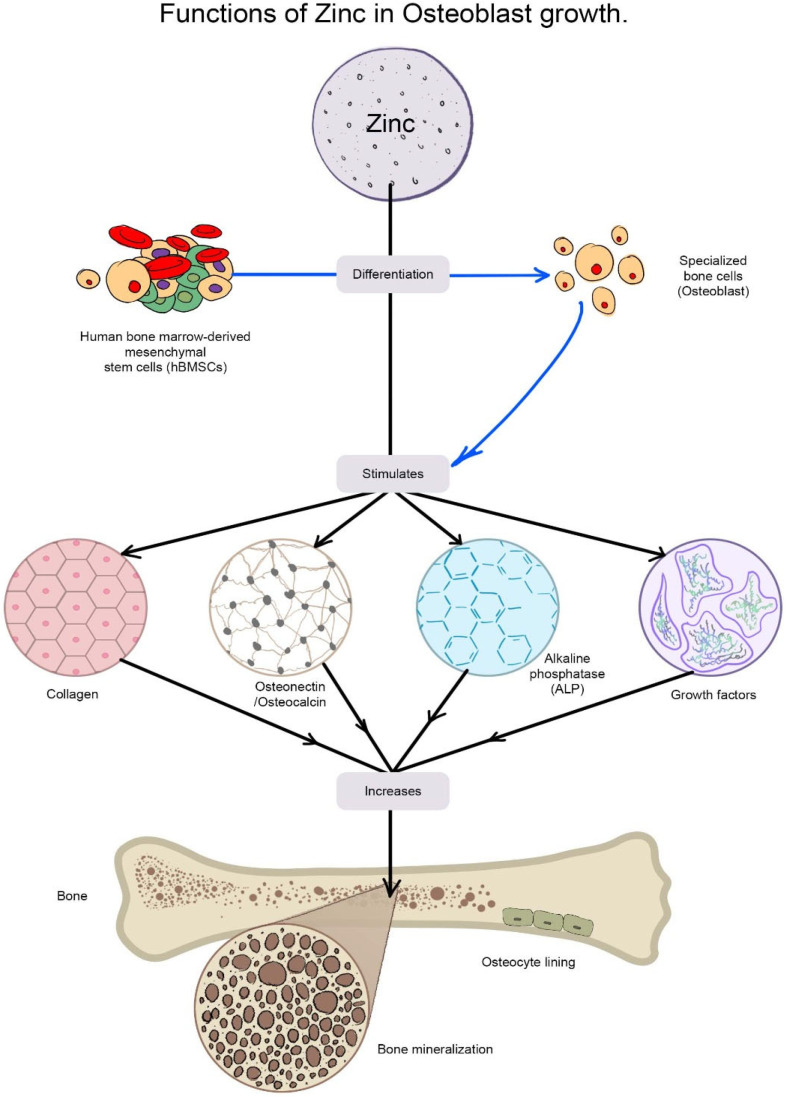
The diagram shows the functions of Zn in stimulating osteoblastic bone formation and mineralization. Zinc stimulates gene expression of various proteins including type I collagen, alkaline phosphatase, and osteocalcin in the cells. Zn is also known to increase the production of growth factors such as IGF-I and TGF-β1 in osteoblastic cells.

Zn and ZnO have emerged as a recent alternative among these BMs and are commonly employed in combination with other biomaterials to gain diverse qualities in antibacterial ability, cytocompatibility, and corrosion resistance [80,81] due to their customizable size manipulation from micro to nano [82]. Bone is the principal repository for Zn since it stores about 30% [83], and Zn helps in the maintenance of bone mass [84]. It maintains the shape of cell membranes [83] and is crucial for bone quality. In osteoblastic cells, Zn can directly activate aminoacyl-tRNA synthetase, a rate-limiting enzyme during protein translation [85], accelerate cellular protein synthesis [86] and increase the gene expression of the transcription factor Runx2, which is connected to osteoblast differentiation. Zn also prevents the production of osteoclast-like cells from marrow cells, which minimizes osteoclastic bone resorption [87]. Bone mineralization is aided by the enzyme alkaline phosphatase, which employs zinc as a co-factor [88,89,90]. In an in vitro experiment, Zn doses between 7 and 20 nM enhanced alkaline phosphatase activity, but Zn concentrations over 5 µM decreased alkaline phosphatase activity [88,91,92]. These findings imply that a Zn shortage may affect bone growth by impairing osteoid mineralization or calcified cartilage production linked to endochondral ossification. Many distinct types of skeletal defects in prenatal and postnatal development are linked to Zn deficiency, and a study demonstrated that osteoporotic patients had lower skeletal Zn levels than the control [93]. By promoting collagen production, alkaline phosphatase (ALP) activity, and mineralization of bone nodules, Zn can improve osteogenesis (Figure 6).

Yusa et al. showed that eluted Zn ions from Ti surfaces promoted osteoblast activities in human bone marrow-derived mesenchymal stem cells (hBMSCs) and dental pulp stem cells (hDPSCs) [94]. In both cell types, the eluted Zn ions stimulated the expression of osteoblast marker genes (collagen type I, ALP, and osteocalcin) and calcium deposition. In hDPSCs, Zn ions further stimulated the expression of Runx2, vascular endothelial growth factor A, and transforming growth factor-beta. Additionally, apoptosis rates in MC3T3-E1 cells increased from 7% in normal media to 75% and 90% when the cells were grown in Zn-deficient or Zn-free media, respectively [95]. Numerous studies have shown that increasing ZnO content improved antibacterial capacity [96,97,98], and nanocoating with ZnO may minimize *S. epidermidis* adherence, thus enhancing the efficacy of orthopedic implants [99]. Lin, M.-H. et al. detected that the chitosan/ZnO-NP coating showed 1.2-fold stronger antibacterial activity against *E. coli* than the chitosan coating alone and actively prevented the formation of biofilm [100].

Similar to Zn and ZnO, another degradable metal such as Mg provides similar benefits for tissue healing [101]. Adhikari, U. et al. mimicked the nanostructured architecture and chemical makeup of natural bone tissue matrices with a 3D scaffold made from chitosan, carboxymethyl chitosan, calcium phosphate monobasic, and magnesium oxide. This scaffold also served as a source for soluble metal ions that are beneficial to osteoblast cells and offers a favorable background to promote biomineralization [102]. Pure Mg corrodes too quickly in physiological pH and produces excessive hydrogen gas, which is its biggest drawback; thus, efforts to use the metal oxide coating in orthopedic applications have been limited [101]. In addition, the inclusion of biodegradable ZnO-NPs in polycaprolactone enables the gradual release of zinc, which has the potential to improve mesenchymal stem cell (MSC) differentiation as an added advantage. Although osteogenic differentiation was improved on scaffolds with an increased concentration of ZnO, MSC chondrogenic differentiation was boosted on scaffolds with a reduced proportion of ZnO [103].

### 2.7. Antidiabetic Action of ZnO-NPs

Diabetes is a metabolic disorder characterized by persistent hyperglycemia. Zinc has been discovered to have an important role in the production, storage, and secretion of insulin [104]. Furthermore, it improves insulin signaling through pathways, such as elevated PI3K activity, insulin receptor tyrosine phosphorylation, and the inhibition of glycogen synthase kinase [105]. It has been reported that zinc’s insulin-mimicking activity leads to enhanced lipogenesis and decreased nonesterified fatty acid release from adipocytes [106]. ZnO-NPs are more frequently chosen for antidiabetic effects over other metal nanoparticles because they increase the expression of GLUT-4 and INS genes due to the confluence of factors such as the enhanced cellular permeation of biosynthesized ZnO-NPs, the promotion of glycolysis via hepatic glycogenesis, and the elevation of insulin levels. Moreover, it imposes synergistic effects on the expression and activity of increased glucokinase and the expression levels of IRA and GLUT-2 [107].

A study revealed that zinc combined with insulin acts as an autocrine molecule, increasing GSIS from rat-isolated pancreatic islets [108], and interacts with several components of the insulin transduction system, facilitating glucose metabolism and insulin mRNA expression in hepatic tissue of diabetic rats [109]. In an alloxan-induced diabetic model, rats administered with 96 mg/dL of ZnO-NPs synthesized from the seed extract of *Silybum marianum* L. had considerably lower fasting blood sugar (FBS) levels than rats fed with 117 mg/dL of insulin, 110 mg/dL of zinc oxide, and 120 mg/dL of crude extract, implying the potent antidiabetic activity of ZnO-NPs. Antidiabetic medicinal plants have also been used to synthesize ZnO-NPs and studied for antidiabetic effects, such as *Rheum ribes* [110] and *Cosus igneus* [111]. Similarly, the antidiabetic effect of ZnO-NPs synthesized from the flower extract of *Senna auriculata* [112] and leaf extract of *Andrographis paniculata* was studied in terms of α-amylase inhibitory activity, where it showed a lower IC_50_ value (121.42 µg/mL) than the leaf extract of *A. paniculata* (149.65 µg/mL) and ZnNO_3_ (178.84 µg/mL) [113]. Moreover, the antidiabetic activity of ZnO-NPs synthesized from *Withania somnifera* was monitored in terms of inhibition of α-amylase and α-glucosidase, showing 90% and 95% inhibition, respectively, at 100 µg/mL [114]. According to the findings of these studies, ZnO-NPs have a substantial antidiabetic effect in terms of glucose and insulin levels, glucose tolerance, and diabetic dyslipidemia.

### 2.8. Antioxidant Activity of ZnO-NPs

In the modern world, the ingestion of some oxidized meals is associated with numerous serious ailments, such as hepatomegaly or necrosis of epithelial tissues, because they are capable of producing lipid peroxides and other toxic-free radicals [115,116,117]. Various natural and synthetic antioxidants are utilized to neutralize these damaging free radicals, but they have drawbacks such as high reactivity and toxicity when compared to the nanoparticles synthesized these days [118,119]. Das et al. investigated the antioxidant potential of ZnO-NPs and revealed that the antioxidant activity of ZnO-NPs is due to the transfer of electron density from oxygen to the odd electron located at the nitrogen atom in DPPH (2,2-diphenyl-1-picrylhydrazyl), resulting in a reduction in the intensity of the n→π* transition at the 517 nm wavelength [120].

The previous finding showed that the percentage of inhibition of free radicals by ZnO-NPs on DPPH increases along with that of the concentration, explaining the ZnO-NPs’ promising antioxidant potential [121]. Similarly, the antioxidant activity of ZnO-NPs synthesized using the *Aquilegia pubiflora* leaf extract was monitored through four different assays (total antioxidant capacity—TAC, total reducing power—TRP, free radical scavenging assay—FRSA (DPPH), and Trolox antioxidant assay—ABTS) for a better evaluation, and the obtained results in terms of ascorbic acid equivalent per milligram (µg AAE/mg) were directly proportional to the concentration of ZnO-NPs in each assay [68]. In addition to that, similar studies were carried out using ABTS, DPPH, hydrogen peroxide, and super peroxide scavenging assays, where the DPPH assay exhibited direct dose-dependent behavior and the order of antioxidant activity was as follows: ABTS > DPPH > SOR > H_2_O_2_ [122]. Furthermore, several plant sources such as *Salvia hispanica* [123], *Borassus flabellifer* [124], and *Punica granatum* [125] have been utilized for evaluation of the antioxidant activity of ZnO-NPs. Generally, the antioxidant behavior of ZnO-NPs is due to the reducing ability of NPs and the phytochemicals adsorbed/capped on the surface of ZnO-NPs [126]. This reveals the unparalleled antioxidant capacity of ZnO-NPs.

### 2.9. Antiviral Action of ZnO-NPs

ZnO-NPs have been reported to exhibit significant antiviral activities against a plethora of viruses, such as herpes simplex virus (HSV), human papillomavirus (HPV), human immunodeficiency virus (HIV), hepatitis C and E virus (HCV, HEV), and severe acute respiratory syndrome coronavirus (SARS-CoV) [127]. The mechanism of action underlying the antiviral potency of ZnO-NPs is the stimulation of the innate and adaptive immune response via toll-like receptor signaling pathways and proteins down streaming, which results in the production of pro-inflammatory cytokines that inhibit the virus. Zn^2+^ ions exhibit antiviral properties by preventing infection, inactivating virus adsorption/entry, blocking coating, impeding replication, assembly, and release during the virus’s life cycle, and producing reactive oxygen species [128,129,130,131,132]. Zinc inhibits the entry of viruses and viral polyprotein translation, as well as inhibiting viral RNA-dependent RNA polymerase activity, and has been shown to modulate the host immune response to limit viral replication. It is a mediator in the LPS (bacterial lipopolysaccharide)-induced TLR4 (toll-like receptor 4)-dependent MyD88 (myeloid differentiation primary response protein 88) signaling cascade, which results in early NF-κβ activation (nuclear factor-kappa b). This triggers the production of pro-inflammatory cytokines such as TNF-α (tumor necrosis factor-α), IL-1 (interleukin-1), and IL-6 to increase (interleukin-6), which plays a crucial role in the control of viral pathogens [133,134]. Moreover, ZnO-NPs can absorb UV–Vis light, dissociate water molecules, and release Zn^2+^ ions, generating ROS such as hydrogen peroxide, hydroxyl radicals, and superoxide that disrupt the lipids, proteins, carbohydrates, and DNA of the virus, leading to its death [135]. According to Jana et al., polysaccharide-encapsulated ZnO-NPs showed exceptional antiviral action against human cytomegalovirus (HCMV), with cell survival rates of 93.6% and 92.4% at 400 µg/mL [136]. A survey reported that ZnO-NPs and PEGylated ZnO-NPs have inhibitory effects on the H1N1 influenza virus, with PEGylated ZnO-NPs showing higher anti-influenza activity with less cytotoxicity on MDCK-SIAT1 cells than ZnO-NPs, indicating that PEGylation on the surface of ZnO-NPs enhanced antiviral activity while reducing cytotoxicity [137]. A recent study on ZnO-NPs demonstrated compelling antiviral activity against SARS-CoV-2 at a very low concentration (IC_50_ 526 ng/mL), and it was found that ZnO-NPs can produce a large number of free radicals which ultimately induce significant damage to the membrane proteins of SARS-CoV-2. However, ZnO-NPs displayed cytotoxic levels (CC_50_ 292.2 ng/mL) against VERO-E6 cells [138]. Similarly, they exhibit excellent antiviral activity against the Chikungunya virus [139], and these findings suggest that ZnO-NPs might be good antiviral agents.

### 2.10. Cardioprotective Action of ZnO-NPs

As ZnO-NPs possess potent antioxidant activity, this gives us an idea about their use in the scavenging O_2_^•^— free radicals, which on the other side, possibly have cardioprotective effects. The O_2_^•^— free radicals are produced from lipid peroxides obtained from today’s fast foods and are made up of several flavoring/bleaching agents such as monosodium glutamate (MSG), which have several adverse effects on the heart, liver, kidney, testis, pancreas, brain, and other various tissues and organs with signs of inflammation [140,141,142]. These free radicals must be scavenged using ZnO-NPs to reduce the adverse effects of oxidative stress produced from the heart failure marker, lipid peroxidation (LPO), and lactoperoxidase-like reactive oxygen species free radicals. A study on the alleviation effect of the ZnO-NP/GTE complex on rats, through feeding two dosages of MSG and a dose of ZnO-NP/GTE (10 mg/kg) by oral gavages daily for 30 days, revealed that there was a reduction in LPO markers such as O_2_^•^— free radicals with a significant improvement in the level of endogenous antioxidants such as SOD, CAT, GSH, and GPx in cardiac tissue, indicating the protection against oxidative stress [143]. Thus, ZnO-NPs are believed to restore abnormal cardiac myofiber, implying their cardioprotective potential.

### 2.11. Anthelminthic Action of ZnO-NPs

ZnO-NPs have a strong anthelminthic effect, which is achieved by inducing oxidative stress by producing hydroxyl ions and ROS, which induces helminth membrane damage by electrostatic binding [144,145]. An in vitro study of ZnO-NPs on *Gigantocotyle explanatum* [146] revealed that they possess effective anthelminthic properties in higher concentrations. Flukes survive at lower quantities by increasing the activity of their intracellular antioxidant enzymes, SOD and GST, which scavenge reactive oxygen species [147], whereas with higher concentrations, SOD and GST possibly become saturated due to overproduction of ROS and hydroxyl ions, which leads to detoxification in flukes. These findings demonstrate sufficient evidence for the anthelminthic potential of ZnO-NPs.

## 3. Approaches for Synthesizing ZnO-NPs

**Figure 7:**
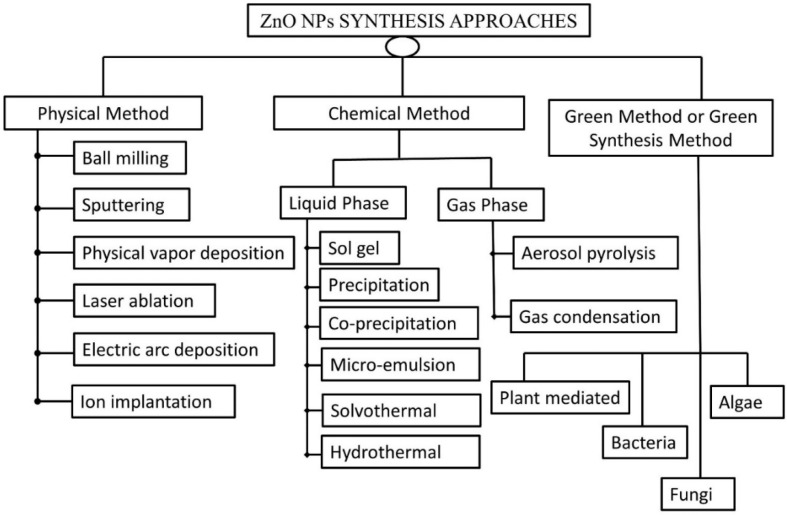
Synthesis approaches for ZnO-NPs.

ZnO-NPs are typically synthesized by utilizing physical, chemical, and biological processes that utilize either top-down or bottom-up approaches (Figure 7). The cutting, grinding, or attrition of larger particles, followed by the formation of smaller particles at the nanoscale level, is referred to as a top-down technique. This method is commonly used for nanoparticle synthesis on a small scale [148]. The bottom-up approach is the process of synthesis of nanoparticles by gathering already miniaturized atoms/molecules through the application of chemical and physical methods. It is a cheaper method and faster than the top-down approach [149].

### 3.1. Physical Methods

Physical methods are used to synthesize ZnO-NPs by attracting smaller molecules and atoms to produce nanoscale-sized particles that employ physical forces. Physical methods comprise ball milling, sputtering, physical vapor deposition, laser ablation, ion implantation, and electric arc deposition. Ball milling is a nonequilibrium phenomenon in which materials of a larger size are crushed with a ball mill due to collision with high-energy balls. The ball milling process has efficient production rates and is easier and more cost-effective. Salah et al. suggested that 15 spherical balls with a circumference of 20 mm concealed in a 500 mL bowl be used to form nanostructures of ZnO in a study on the antibacterial effectiveness of ZnO-NPs [149]. Laser ablation methods refer to the process of the removal of particles from the solid and liquid interface using a laser beam as an energy source. A study conducted by Mintcheva et al. provides a piece of evidence that the millisecond-pulsed laser ablation technique produced rod-shaped ZnO-NPs with lengths ranging from 40 to 110 nm and an average diameter of 30 nm [150]. Physical vapor depositions are a frequently used method in which the deposition of metals coating the surface involves two phenomena, such as evaporation and sputtering. Sputtering is the process of expelling particles from the surface by impacting high-energy particles with plasma ions [151]. Thermal evaporation is another physical approach in which powdered or condensed products are heated to a higher temperature, evaporation occurs, and the resulting vapors condense to form desirable nanoparticles under controlled conditions such as pressure, temperature, humidity, substrate, and so on [152].

### 3.2. Chemical Methods

The chemical methods for synthesizing ZnO-NPs are categorized based on their physical state, which includes solid-phase, liquid-phase, and gas-phase synthesis. Liquid-phase synthesis is a widespread method and a viable alternative to gaseous-phase synthesis. For liquid-phase synthesis, the sol-gel process, colloidal methods, precipitation and co-precipitation methods, microemulsion method, hydrothermal synthesis, and solvothermal and sonothermal methods can be utilized, whereas inert gas condensation methods and pyrolysis can be used for vapor-phase synthesis [153].

#### 3.2.1. Liquid-Phase Synthesis

The sol-gel process is the process of conversion of prepared colloidal solution (sol) into gel through hydrolyzation, condensation, and polymerization reactions. Zinc acetate hydrate in alcohol is the most used precursor for the synthesis of ZnO-NPs [154]. Khan and companions synthesized pure and uniform thorn-like ZnO-NPs of a size < 50 nm for the first time by the sol-gel method [155]. Similarly, precipitation and co-precipitation methods involve the formation of a precipitate when inorganic alkalis act as a reducing agent combined with zinc salt. Sodium hydroxide and zinc sulfate heptahydrate are used as precursors, and by adjusting reaction conditions, these precipitates were washed and calcined at the requisite temperature to produce nanoparticles with the desired shape, size, and characteristics [156].

Solvothermal synthesis is a technique for facilitating a precursor interaction during synthesis by utilizing a solvent at moderate to high pressure (1–10,000 atm) and temperature (100–1000 °C) [157]. Hydrothermal synthesis, on the other hand, employs water and is normally performed below the supercritical temperature of the water, i.e., 374 °C. The microemulsion is another technique of synthesizing the thermodynamically stable dispersion of two immiscible liquids, namely, water and hydrocarbons. In general, two forms of microemulsions are utilized, such as oil-in-water (O/W) and water-in-oil (W/O), with the latter being predominantly used for the preparation of NPs by dispersing the metal salt (Zinc salt) precursor in the aqueous phase. Surfactant- and co-surfactant-charged hydrophilic groups aid to minimize interfacial tension between two phases and enhancing colloidal stability [158].

#### 3.2.2. Gas-Phase Synthesis

The aerosol pyrolysis method is the most commonly used gas-phase synthesis method, in which aerosol droplets dispersed in the gas phase generate aerosol droplets of the precursor zinc salts when heated in a flame. The flame heating causes dehydration, which helps to reduce the size of particles in the nanoscale. The required material decomposes and sinters as a result of the heating over the flame [159]. Inert gas condensation is another major gas-phase synthesis technique. It involves evaporating zinc inside a heat-resistant compartment using a variety of heat sources, such as electron and laser beams or radio frequencies, and then condensing the vapors by migrating them to cooler chambers containing inert gas. Based on the catalyst, this approach is divided into two categories: physical vapor deposition intrigued without catalytic contact and chemical vapor deposition fascinated with catalytic interaction. It may cause agglomeration and coalescence of nanoparticles, which is a typical demerit of this process. Uhm and coworkers synthesized ZnO-NPs of a better shape and size with a 30 nm diameter by the levitational gas condensation method [160].

### 3.3. Green Synthesis

The terms “biological synthesis” and “green synthesis” are often used interchangeably. However, for a biological synthesis to be green, it should comply with the basic principles of green chemistry such as being environmentally friendly, no use of toxic chemicals, reduced derivatization, energy consumption, waste, and so on [161]. Here, green synthesis is the process of synthesizing nanoparticles by incorporating mainly cell extracts (microbial, plant, fungus, algae, etc.) into the substrate involving biofabrication, i.e., the capping of nanoparticles from natural products such as phytochemicals from plants and proteinous extracts from microorganisms and fungus without using any toxic chemicals. Green synthesis is to be nonhazardous, aligning with the principles of green chemistry. These methods provide merits of biocompatibility, cost-effectiveness, large-scale productivity, ecofriendliness, and being devoid of hazardous chemicals and adverse reaction conditions and are, therefore, an attractive alternative to traditional physical and chemical methods [162]. As such, microbial and plant extracts release phytochemicals that act as reducing agents as well as fabricating or stabilizing agents; this eliminates the dependence on industrial chemicals. On the contrary, if synthetic chemicals/solvents are employed to assist the reduction-stabilization process or to maintain pH in a green synthesis, such synthesis is better described as biochemical synthesis.

#### 3.3.1. Plant-Mediated Synthesis of ZnO-NPs

A multitude of research supports the synthesis of crystalline ZnO-NPs by chelating a zinc complex with plant extracts. The aerial parts of plants, such as leaves and flowers, are commonly used in green synthesis. To optimize ZnO-NP synthesis, usually, reaction parameters such as temperature, pH, concentration, and time are adjusted. The appearance of a yellow coloration generally indicates the formation of ZnO-NPs, which is further confirmed by qualitative investigations such as UV–visible spectroscopy, SEM, and TEM [163].

The synthesis of ZnO-NPs with regulated shapes and sizes was accomplished by varying the concentration of plant extracts. Madan et al. synthesized NPs of varied sizes ranging from 9–40 nm and different shapes such as bud, cone, closed pine cone, bullet, and hexagonal disk by altering the concentrations of a plant extract from the leaves of *Azadirachta indica* [164]. The possible mechanism of the green synthesis has been explained by several researchers and the result is that the secondary metabolites and proteins present in the plant extracts act as capping and reducing agents which promote nanoparticle synthesis, whereas some studies have proposed that the nanoparticles of metal ions are formed due to the electrostatic interaction of plant proteins and metal ions. Proteins would reduce the metal ions, resulting in a change in the protein secondary structure, as well as in the formation of metal oxide nanoparticle seeds [163,165]. Plant components, from leaf to root, are extensively utilized in metal oxide nanoparticle synthesis because phytochemicals such as polyphenolic compounds, vitamins, polysaccharides, amino acids, alkaloids, terpenoids, etc. extracted from plants aid in the efficient bioreduction of metal ions for the synthesis of NPs that are stable and variable in structure and dimension. Bioreduction is the process of reducing metal ions or metal oxides to zero-valence metal NPs, fascinating in maintaining their stability. These techniques yield a large quantity of very pure nanoparticles that are free of contaminants [166,167]. Table 1 summarizes the key findings of extensive research on several plants employed in the synthesis of ZnO NPs.

#### 3.3.2. Green Synthesis Using Bacterial Extracts

The nanoparticle synthesis using bacterial extracts is a complex and time-consuming technique of green synthesis. It is vital to ensure vigilant monitoring of the culture media throughout the process to avoid contamination. Otherwise, synthesized NPs could be less optimized and ineffective [2]. A study reported that the synthesis of ZnO-NPs can be carried out using *Rhodococcus pyridinivorans* and zinc sulfate as the substrate. The synthesized NPs were spherically shaped with a 100–130 nm size range confirmed through FE-SEM and XRD analysis [181]. The synthesis of nanoflowers (40 nm width and 400 nm height) with potent photocatalytic potency was also performed with *B. licheniformis* using the green synthesis technique [182]. The excellent antioxidant activity of NPs synthesized using *Pseudomonas aeruginosa* was also revealed, indicating that enhanced NP stability was attained due to the rhamnolipid of bacteria used. Thus, it is significant to consider that bacteria can be used as a better capping agent with outstanding stability and potency [183]. Green synthesis using a bacterial strain is well illustrated in Table 2.

#### 3.3.3. Green Synthesis Using Fungal Extracts

Due to the efficient and large-scale productivity, lower cost, and convenient processing, numerous fungal strains are being used for the green synthesis of ZnO-NPs over bacteria [2]. Fungi are more tolerable and have better metal bioaccumulative properties than bacterial strains, making them a stronger candidate for nanoparticle synthesis [191]. A study found that fungal strains such as *Candida albicans* could be employed to synthesize quasispherical-shaped ZnO-NPs [192]. Similarly, the mycelia of *Aspergillus fumigatus* were used to make spherical aggregate-shaped NPs, which agglomerate into a larger size after a few days, indicating the stability and potent capping activity of fungus as a substrate [193]. Some examples of fungal-mediated synthesis are included in Table 3.

#### 3.3.4. Green Synthesis Using Microalgae and Macroalgae

Algae are photosynthetic organisms that are made up of single or multiple cells and lack essential components such as roots, stems, and leaves. Algae are classified into two types, macroalgae, and microalgae, as well as three groups, Rhodophyta (red pigmented), Phaeophyta (brown pigmented), and Chlorophyta (green pigmented). Algae have a limited significance in the synthesis of ZnO-NPs and are better suited for the production of other metal nanoparticles such as silver and gold nanoparticles. Microalgae are commonly employed for the green synthesis of NPs because they have a greater potential to minimize metal toxicity through the biodegradation process [198]. ZnO-NPs are typically synthesized using algae from the Sargassaceae family. *Sargassum muticum* was employed to make hexagonal wurtzite-shaped ZnO-NPs [199]. Similarly, nanoparticles of spherical, radial, triangular, hexagonal, and rod shapes were synthesized from *S. myriocystum* [200]. Furthermore, *Chlamydomonas reinhardtii*, a species of the Chlamydomonaceae family, was used to synthesize various-shaped NPs, such as nanorods, nanoflowers, and porous nanosheets [201]. Table 4 summarizes the ZnO-NPs synthesized by some of the algae.

## 4. Characterization of ZnO-NPs

**Figure 8:**
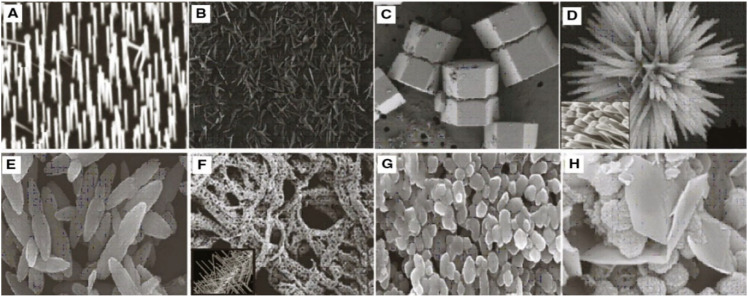
Morphology of ZnO nanostructures: (**A**) needles, rods, and wires; (**B**) helixes and springs; (**C**) nanopellets/nanocapsules; (**D**) flower, snowflake, and dandelion; (**E**) peanut-like; (**F**) interwoven particle hierarchy; (**G**) raspberry, nanosheet/nanoplate; (**H**) circular/round or sphere-shaped. (Reprinted from [209]; open access under CC BY).

A plethora of studies suggests that the morphology and surface chemistry of nanoparticles influence their biodistribution, safety, and effectiveness in biological systems (Figure 8). Characterization is the core tool for successful applications and the understanding of nanoparticles. Nanoparticle size characterization is complicated by the polydispersity of materials, yet it is important to determine the morphology since the nanoparticle size’s resemblance to biological moieties is assumed to impart many of their distinct nanomedicine capabilities. Optical microscopy cannot resolve nanostructures; therefore, electron microscopy is used to characterize the nanoparticles. SEM and TEM are used to characterize the shapes and sizes, but TEM is used more often because it uses more powerful electrons and presents high resolution and informative image details regarding the atomic scale-like morphology, aggregation state, and distribution, and observes the functionality of capping agents/phytochemicals in enclosing NPs. Some biological molecules such as liposomes and proteins do not deflect the electron beam sufficiently and are invisible to electromagnetic radiation; therefore, dynamic light scattering (DLS), a nondestructive approach that uses a monochromatic laser and is also known as photon correlation spectroscopy, is used to characterize these compounds in suspensions and solutions. Here, small changes in the intensity of scattered laser light in the nanoparticle solution are regulated with a photon detector to analyze the hydrodynamic diameter and morphology of NPs [204].

The characterization of nanoparticles in animal tissue is accomplished by energy dispersion X-ray analysis (EDX), which assists in identifying the elemental composition and linkage of metabolites and also facilitates the interpretation of biodistribution of synthesized nanoparticles. Furthermore, atomic force microscopy (AFM) helps in determining the 3D geography (height and volume) of NPs; Fourier transform infrared spectroscopy (FTIR)-attenuated total reflectance (ATR) is an easy and nondestructive technique that contributes metabolites, chemicals, etc. through the synthesis and capping of NPs; UV–visible-diffuse reflectance spectroscopy (UV-DRS) is used to study the optical property of colored samples where the reflectance measurements are utilized to investigate the surface plasmon resonance of metals and hypersensitive biological analysis [205]; thermal gravimetric-differential thermal analysis (TG-DTA) provides information about the thermal stability, phase transition, and effect of the oxidative as well as reductive environment; photoluminescence (PL) analysis is utilized to determine the band gap, and crystalline purity and impurities; and x-ray photoelectron spectroscopy (XPS) can be used to characterize the morphology, and bioactive surface and material surface chemistry of NPs [206,207,208].

ZnO is one of the most significant II-VI compound semiconductor materials in scientific research and technological applications with noncentrosymmetric structures and multiple shape-induced functions. By adjusting the hydrothermal reaction parameters (such as precursor concentration, reaction duration, and pH), several morphologies of ZnO, including microrods, hexagonal pyramid-like rods, and flower-like rod aggregates, have been synthesized, respectively, on glass substrates. The production of ZnO microrods is significantly influenced by the precursor concentration. With longer reaction times, ZnO crystals can change from hexagonal pyramids to rod-like laths. ZnO rod aggregates that resemble flowers are produced at higher pH levels. The findings could provide a strategy for producing ZnO crystals in a certain desirable form [210]. Similarly, in a recent study, Doustkhah et al. hydrothermally transformed zinc-based metal-organic frameworks into ZnO nanostructures with temperature-dependent tunable structures and catalytic activity, which at an elevated temperature displayed high crystallinity and better dye degradation efficiency than at a lower temperature [211].

Most of the group II-VI binary compound semiconductors crystallize as hexagonal wurtzite or cubic zinc-blende, with each anion surrounded by four cations at the corners of a tetrahedron. The iconicity of the II-VI compound semiconductor ZnO lies at the interface between covalent and ionic semiconductors. Wurtzite, blende, and rocksalt are potential ZnO crystal formations. Wurtzite is the most thermodynamically stable of these crystal forms at room temperature, but blende is stable when developed on a cubic substrate and rocksalt is stable when synthesized at very high temperatures [212]. In contrast to the zinc-blende structure, which has two interpenetrating face-centered-cubic (fcc) sublattices that are displaced along the body diagonal by one-quarter of a body diagonal, the wurtzite structure is made up of two interpenetrating hexagonal-closed-packed (hcp) sublattices. Due to the decrease in lattice dimensions, which favors iconicity over a covalent nature, and the structure’s six-fold coordination, wurtzite can undergo the same transformation as other II-VI semiconductors to become rocksalt [212].

## 5. Conclusions

This review aimed to explore the synthesis, characterization, and biological activities of ZnO-NPs, illustrating their mechanism of action. Extensive discussion was centered on the green synthesis approach and its biomedical applications. The pathways of different bioactivity were explained, with special emphasis on ZnO-NPs’ biopotency with regard to antibacterial, antifungal, anticancer, anti-inflammatory, antidiabetic, antioxidant, antiviral, wound healing, orthopedic implants, bone healing, and cardioprotective activity, along with the concise interpretation of the green synthesis of nanoparticles using biological sources. The importance and significance of ZnO-NPs in pharmaceutical and biological sectors have attracted scientists to perform an extensive study of their applications in multiple ailments. Green synthesis is an eco-friendly approach that reduces costs, increases production, and improves biocompatibility in humans. Biofabrication with natural compounds helps to stabilize the nanoparticles with reduced toxicity and higher reduction potential. ZnO-NPs possess several compelling pharmacological activities. Special focus should be given to ZnO-NP generation through plant-mediated synthesis, bearing tremendous applications in the fields of pharmaceuticals, food, and cosmetics. The advancement of nanotechnology in the formulation of metal oxide nanoparticles can contribute to the reduction in the dosage used with optimum desired effects and low toxicity.

## Figures and Tables

**Table 1:** Summary of the plant-mediated synthesis of zinc oxide nanoparticles.

Biological Source	Used Plant Parts	Extraction Technique	Zinc Precursors; Condition	Size of Nanoparticles Synthesized (nm)	Morphology of Nanoparticles	References
*Albizia lebbeck*	Stem bark	Decoction at 60 °C	Zinc nitrate hexahydrate and sodium hydroxide, calcined at 350 °C	DLS: 82.31 at 0.05 molar and 110 at 0.01 molar SEM: 66.25, 82.52, 112.87 at 0.1, 0.05, and 0.01 molar concentration	Rod and hexagonal	[168]
*Abutilon indicum*	Leaf	Solvent extraction at 90–95 °C	Zinc nitrate hexahydrate	XRD: 16.72	Spheroid or rodlike	[169]
*Azadirachta indica*	Leaf	Soxhlet extraction at 350 °C	Zinc nitrate	XRD: 11–40	Hexagonal disk	[164]
*Berberis aristata*	Leaf	Boil	Zinc acetate dehydrate, sodium hydroxide	XRD: 5–25 DLS: 90–110	Needle	[170]
*Camellia sinensis*	Solid waste	Decoction	Zinc acetate, pH 12	XRD: 19.5	Rod	[171]
*Cassia fistula*	Leaf	Decoction at 70 °C	Zinc acetate dihydrate; 70 °C	XRD: 2.72 DLS: 68.1	Spherical	[172]
*Citrus limon*	Leaf	Decoction at 60 °C	Zinc nitrate	TEM: 37.05 ± 18.27 DLS: 50.8	Spherical	[173]
*Crotalaria verrucosa*	Leaf	Boil	Zinc nitrate hexahydrate	TEM: 27 XRD: 17.47 DLS: 27	Hexagonal wurtzite	[174]
*Limonia acidissima*	Leaf	Decoction at 60 °C	Zinc nitrate: pH 10	HRTEM: 12–53	Spherical	[175]
*Melia azadarach*	Leaf	Decoction at 70 °C	Zinc acetate dihydrate; 70 °C	XRD: 2.72 DLS: 3.62	Spherical	[172]
*Mentha pulegium*	Leaf	Boil	Zinc nitrate hexahydrate	TEM: 40 FE-SEM: 38–49 XRD: 44.94	Hexagonal, quasispherical	[176]
*Mussaenda frondosa*	leaf, callus, and stem	Reflux at 100 °C	Zinc nitrate hexahydrate, calcined at 400 °C	XRD L-ZnO-NP: 8 and 15 C-ZnO-NP: 5 and 7 S-ZnO-NP: 9 and 12	L-ZnO-NP: hexagonal wurtzite C-ZnO-NP and S-ZnO-NP: spherical	[177]
*Myristica fragrans*	Fruit	Decoction at 150 °C	Zinc acetate dihydrate; calcined at 500 °C	TEM: 35.5 SEM: 43.3–83.1 XRD: 41.23	Spherical and hexagonal	[178]
Oats	Oat biomass	Boil	Zinc nitrate hexahydrate, calcined at 400 °C	DLS, SEM, TEM: 100 XRD: 17.52	Wurtzite and hexagonal	[179]
*Tabernaemontana divaricata*	Leaf	Decoction at 80 °C	Zinc nitrate hexahydrate at 450 °C	TEM: 20–50 XRD: 36.82	Hexagonal wurtzite	[180]

**Table 2:** Summary of the bacteria-mediated synthesis of zinc oxide nanoparticles.

Strain of Bacteria	Family	Size of Nanoparticles Synthesized (nm)	Morphology of Nanoparticles	References
*Rhodococcus* *pyridinivorans*	Nocardiaceae	FE-SEM: 100–120 XRD: 120–130	Hexagonal phase and roughly spherical	[181]
*Pseudomonas* *aeruginosa*	Pseudomonadaceae	TEM: 35–80 XRD: 27, DLS: 81	Spherical	[183]
*Pseudomonas* *aeruginosa* NMJ15	Pseudomonadaceae	TEM: 6–21 XRD: 21	Spherical	[184]
*Aeromonas* *hydrophila*	Pseudomonadaceae	AFM: 57.72 XRD: 42–64	Oval and spherical	[185]
*Lactobacillus sporogens*	Bacillaceae	TEM: 5–15 XRD: 11	Hexagonal	[186]
*B. licheniformis*	Bacillaceae	TEM: 200 (nanopetal 40 nm width and 400 nm length)	Nanoflower	[182]
*Serratia ureilytica* (HM475278)	Enterobacteriaceae	SEM: 170–250 (at 30 min), 300–600 (at 60 min), 185–365 (at 90 min)	Spherical and nanoflower	[187]
*Arthrospira platensis*	Microcoleaceae	TEM: 30–55 XRD: ≈45	Spherical	[188]
*Desertifilum* sp. EAZ03	Desertifilaceae	TEM: 88 XRD: 60–80	Rod	[189]
*Marinobacter* sp. 2C8 and *Vibrio* sp. VLA (cell-free extract)	Alteromonadaceae Vibrionaceae	2C8-TEM: 10.23 ± 2.48 VLA-TEM: 20.26 ± 4.44	Hexagonal wurtzite	[190]

**Table 3:** Summary of the fungal-mediated synthesis of zinc oxide nanoparticles.

Fungal Strain	Family	Size of Nanoparticles Synthesized (nm)	Morphology	References
*Aspergillus niger*	Trichocomaceae	SEM: 61 ± 0.65 XRD: 41	Spherical Crystalline wurtzite	[194]
*Candida albicans*	Saccharomycetaceae	XRD: 25, SEM: 15–25, TEM: ~20	Hexagonal wurtzite, quasispherical	[192]
*Aspergillus fumigatus TFR-8*	Trichocomaceae	DLS: 1.2–6.8	Oblate spherical and hexagonal	[195]
*Aspergillus strain*	Trichocomaceae	SEM: 50–120	Spherical	[196]
*Xylaria acuta*	Xylariaceae	TEM: 30–50, average: 34 SEM: 40–55 DLS: 30–50 XRD: 35–45	Rod and hexagonal	[197]

**Table 4:** Summary of the algal-mediated synthesis of zinc oxide nanoparticles.

Algae Strain	Family	Size of As-Synthesized Nanoparticles (nm)	Morphology of the Nanoparticles	Surface Functional Groups	References
*Sargassum* *muticum*	Sargassaceae	FE-SEM: 30–57 XRD: 42	Hexagonal wurtzite	Sulfate group asymmetric with stretching band, asymmetric C–O band coupled with C-O-SO_3_ and -OH group, sulfated polysaccharides	[199]
*Sargassum muticum*	Sargassaceae	SEM: 50 DLS: 25–50 XRD: 15–50	Spherical	3432 and 1609 cm^−1^ presence of O–H stretching, 500 cm^−1^ below suggests a Zn–O stretching vibration	[202]
*Chlamydomonas* *reinhardtii*	Chlamydomonaceae	HR-SEM: 55–80 XRD: 21	Rod	N–H bending band of amide I and amide II, C=O stretching of zinc acetate C=O, and C–O–C stretch of polysaccharide	[201]
*S. myriocystum*	Sargassaceae	DLS: 46.6 AFM: 20–36 TEM: 76–186	Rectangular, triangle, radial hexagonal, rod, and spherical shape	Carboxylic acid, with O–H and C=O stretching bands	[200]
*Ulva lactuca*	Ulvaceae	TEM: 10–50, av.: 15 XRD: 5–15	Triangle, hexagon, rod	420 cm^−1^ suggests ZnO, peaks at 1634.00, and 620.93 cm^−1^ suggests ZnO stretching and deformation vibration	[203]

## Data Availability

All data generated or analyzed during this study are available within the article.

## Abbreviations

**ZnO-NPs:** zinc oxide nanoparticles; **ROS:** reactive oxygen species; **SOD:** superoxide dismutase; **GSTs:** glutathione S-transferases; **NPs:** nanoparticles; **SEM:** scanning electron microscopy; **TEM:** transmission electron microscopy; **XRD:** X-ray diffractometer; **DLS:** dynamic light scattering; **EM:** electron microscopy; **HRTEM:** high-resolution transmission electron microscopy; **HRSEM:** high-resolution scanning electron microscopy; **FE-SEM:** filed emission scanning electron microscopy; **AFM:** atomic force microscopy; **GSH:** glutathione; **GPx:** glutathione peroxidase; **MSG:** monosodium glutamate; **DPPH:** 2,2-diphenyl-1-picrylhydrazyl; **NEFA:** nonesterified fatty acid; **iNOS:** inducible nitric oxide synthase; **PGE2:** prostaglandin E2; **NF-κβ:** nuclear factor-kappa b; **COX 2:** cyclooxygenases 2; **IL-1:** interleukin-1; **TNF:** tumor necrosis factor; **IL-6:** interleukin-6; **IL-12:** interleukin-12; **IL-18:** interleukin-18; **ATR:** attenuated total reflection; **EDAX:** energy dispersion analysis of X-ray; **PL:** photoluminescence; **XPS:** X-ray photoelectron microscopy; **TG-DTA:** thermal gravimetric-differential thermal analysis; **UV-DRS:** UV–visible reflectance spectroscopy; **BMs:** biodegradable metals; **ALP:** alkaline phosphatase; **hBMSCs:** human bone marrow-derived mesenchymal stem cells; **hDPSCs:** human dental pulp stem cells; **MDR:** multidrug-resistant; **HPMC:** hydroxypropyl methylcellulose; **FBS:** fasting blood sugar; **CAT:** catalase.
